# Infrared Ion Spectroscopic Characterization of the Gaseous [Co(15-crown-5)(H_2_O)]^2+^ Complex

**DOI:** 10.1021/acs.jpca.3c04241

**Published:** 2023-08-18

**Authors:** Musleh Uddin Munshi, Giel Berden, Jos Oomens

**Affiliations:** † Department of Chemistry, Sogang University, Seoul 04107, Republic of Korea; ‡ FELIX Laboratory, Radboud University, Institute for Molecules and Materials, Toernooiveld 7, 6525 ED Nijmegen, The Netherlands; § University of Amsterdam, Science Park 904, 1098XH Amsterdam, The Netherlands

## Abstract

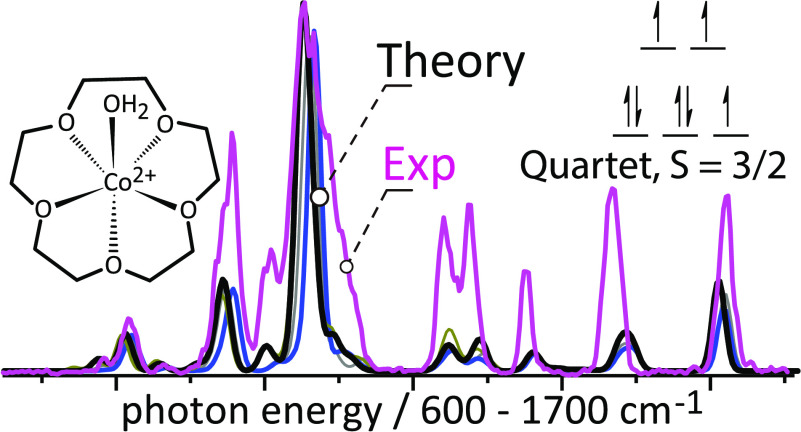

We report fingerprint infrared multiple-photon dissociation spectra of the gaseous monohydrated coordination complex of cobalt(II) and the macrocycle 1,4,7,10,13-pentaoxacyclopentadecane (or 15-crown-5), [Co(15-crown-5)(H_2_O)]^2+^. The metal–ligand complexes are generated using electrospray ionization, and their IR action spectra are recorded in a quadrupole ion trap mass spectrometer using the free-electron laser FELIX. The electronic structure and chelation motif are derived from spectral comparison with computed vibrational spectra obtained at the density functional theory level. We focus here on the gas-phase structure, addressing the question of doublet versus quartet spin multiplicity and the chelation geometry. We conclude that the gas-phase complex adopts a quartet spin state, excluding contributions of doublet species, and that the chelation geometry is pseudo-octahedral with the six oxygen centers of 15-crown-5 and H_2_O coordinated to the metal ion. We also address the possible presence of higher-energy conformers based on the IR spectral evidence and calculated thermodynamics.

## Introduction

Metal cations complexed with crown ethers represent model host–guest systems of high interest in the field of molecular recognition.^1^ Ever since their discovery,^2^ metal complexation of cyclic polyethers received much attention, because of their relevance in fields ranging from industrial to biochemical and medical applications. Examples are the extraction and sensing of dissolved chemicals,^3−5^ water purification^6,7^ through supramolecular complexation,^8,9^ removal of carcinogenic/toxic radio-isotopes from nuclear waste,^10−13^ transporting cytolytic radioactive chemicals near tumors,^14^ designing advanced analytical methods,^11^ and as catalyst in organic synthesis.^15^ Studies on these complexes span the periodic table from alkali,^3,16−18^ alkaline earth,^19,20^ and first row transition metals^21^ to the lanthanides and actinides.^2,3^ The majority of experimental and theoretical studies has probably been devoted to investigations of model systems mimicking biological systems, for instance, the porphyrin ring in the haem protein, ionphores,^22^ cyclic complex in chlorophyll, and the Corrine ring in vitamin B_12_ to disclose structure–function relationships.^15,23,24^

In addition to nuclear magnetic resonance (NMR) and infrared (IR) spectroscopy, circular dichroism (CD) spectroscopy is often employed in studies characterizing these coordination complexes, exploiting the optical isomerism in solution.^25^ Moreover, significant efforts were devoted to theoretical modeling, for instance to better understand the intrinsic *d-d* transitions in chiral transition-metal complexes.^25−28^ Development of theoretical models strongly relies on the availability of experimental data, preferably on model systems with known absolute configurations and in complete isolation.^29,30^

Ion storage mass spectrometry (MS) offers platforms for the study of gaseous charged metal–ligand complexes^31^ in complete isolation, without influence from solvent or solid environment. More than two decades ago, threshold collision-induced dissociation (TCID) MS was employed to determine bond dissociation energies (BDE) of alkali metal ion bound crown ether (e.g., 15-crown-5) complexes in a guided ion beam tandem MS apparatus.^32,33^ Deviations in the BDE were noted between measurement and theory because of the lack of isomer selectivity, and the speculative presence of higher-energy conformers in the MS instrument.^32,33^ Nearly a decade ago, similar experiments were repeated using soft electrospray ionization (ESI) as a source on the same MS.^18^ Based on the newly measured BDE, these studies confirmed^17,18,34^ that excited conformers were accessed.^32,33^ A theoretical explanation was provided as to how the higher-energy conformers could be formed.^17,18^

IRMPD spectra were recorded of mass (*m*/*z*) selected Zn and Cd dications complexed with various crown ethers, including 15-crown-5, which suggested the presence of some slightly higher-energy conformers along with the minimum-energy isomer.^35^ Employing the same technique, Martinez-Haya and coworkers^19,20^ investigated complexation of alkaline-earth metal cations (Mg^2+^, Ca^2+^, Sr^2+^, and Ba^2+^) with 18-crown-6. Their investigation showed that these metal ions form similar but more tightly bound complexes than alkali metals.^19^ These metal cations formed hexa-coordinated complexes, where the metal ion is chelated inside the folded macrocycle. The alkali and alkaline-earth metal ions formed equatorial chelation complexes with the 18-crown-6 ligand, clearly distinguishable from an octahedral geometry according to IRMPD spectroscopy and theoretical calculations. Recently, we investigated complexation of Ni^2+^ with hexacyclen (the nitrogen analog of 18-crown-6) by recording the IRMPD spectrum; theoretical interpretation confirmed the dominant occurrence of pseudo-octahedral meridional stereoisomers^25,36−38^ with high-spin state in the gas phase.^39^ Note that complexation of Co^3+^ with hexacyclen showed a similar isomer distribution in solution.^36^

Like spin crossover complexes of Fe(II),^40−42^ low- and high-spin configurations have been reported for six-coordinate Co(II).^43−46^ The [CoN_6_]^2+^ (iodide salt) derivatives of bidentate and tridentate ligands were examples of such equilibrium spin mixtures at room temperature.^47^ Note that fivefold-coordinated Co(II) complexes were also shown to display such behavior.^48,49^ These ionic complexes exhibit characteristic magnetic properties^50^ due to the number of unpaired electrons in the *d*-shell, which has been employed to assign the spin multiplicity, including corresponding geometries. In general, these complexes are known to show variations of the metal–ligand bond lengths when swapping between spin multiplicities (i.e., doublet ↔ quartet for Co^2+^).^46^ Low-spin configurations often show noticeable Jahn–Teller effect due to the single electron in an e_g_ orbital.^47,51^ Furthermore, spin states are also influenced by the type of ligands,^46^ temperature, and solvent.^47,52^ Although challenging,^47^ measuring the magnetic moment often as a function of temperature remains the principal technique. On the contrary, it would be worthwhile to address the structural characterization using IR spectroscopy because of its sensitivity toward molecular geometry.

**Scheme 1:**
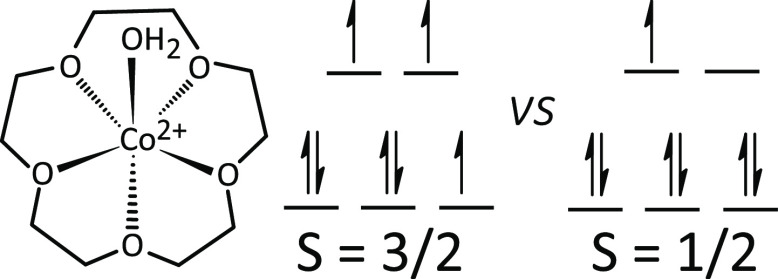
Hydrated Co(II) Complex [Co(15-Crown-5)(H_2_O)]^2+^ and Possible Occupancies of the Five Co 3d Orbitals Giving Rise to Either Quartet or Doublet Spin Multiplicities

Studies have been reported of hydrated alkali metal ion complexation with 18-crown-6^22^ and [Mn(II)(benzo-15-crown-5)(H_2_O)_0–2_]^53^ in the gas phase. Gas-phase data is far more scarce for Co(II) than for Fe(II), especially in an octahedral ligand environment. It often remains challenging to accurately model structural and corresponding spectroscopic properties of transition metal–ligand complexes,^54−56^ due to the near-degeneracy of the partially occupied *d*-orbitals.^57^ Co^2+^ (d^7^) in an octahedral ligand environment belongs to this paradigm. Although synthesis and structural characterization in condensed phases have been reported,^50,51,58−60^ gas-phase data is scarce. In this study, we investigate monohydrated Co^2+^ complexed with a crown ether, [Co(15-crown-5)(H_2_O)]^2+^ (see Scheme 1) in complete isolation to capture the effect of microsolvation on the structure and IR spectra. We record the IR spectrum of the gaseous mass-to-charge (*m*/*z*) selected complex to characterize electronic and geometric structures. We examine the isolated complex employing IRMPD spectroscopy in a Paul-type quadrupole ion trap (QIT) MS^61,62^ coupled to the beamline of the wavelength-tunable infrared free-electron laser FELIX. Computed vibrational spectra are compared with the action spectra to extract structural information.

## Methods

### IRMPD Action Spectroscopy

Experiments were performed in a modified quadrupole ion trap mass spectrometer (QIT MS, Bruker, AmaZon Speed ETD, Bremen, Germany), which has been described in detail elsewhere.^62^ The cation of interest, [Co(15-crown-5)(H_2_O)]^2+^ at *m*/*z* 148, was generated by electrospray ionization (ESI) starting from a solution containing equimolar (1 μM) amounts of Co(NO_3_)_2_ salt and 15-crown-5 in 1:1 MeOH:H_2_O. IRMPD spectra were recorded from 600 to 1800 cm^–1^ using wavelength tunable infrared radiation from the FELIX free-electron laser (FEL),^63,64^ which was operating at a repetition rate of 10 Hz while producing 6 μs long macropulses with energies up to 90 mJ per pulse.

Mass-selected ions were accumulated for 50 ms followed by irradiation with two FEL macropulses at maximum pulse energy. Trapped ions are excited whenever the laser frequency is in resonance with one of the vibrational absorption bands of the ion. Rapid statistical redistribution of the absorbed energy over all vibrational degrees of freedom promotes the increase of internal energy of the ions *via* intramolecular vibrational redistribution (IVR).^65,66^ Once the accumulating internal energy exceeds the dissociation threshold, the ion can undergo fragmentation. H_2_O loss was the only fragmentation channel observed. IR spectra are generated by plotting the fragmentation yield as a function of laser frequency, where the yield is defined as:^39,67−69^
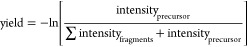


The fragmentation yield was linearly corrected for frequency-dependent variations in the laser pulse energy and a grating spectrometer was used to calibrate the IR frequencies. Wavelength scans were performed with a step size of 3 cm^–1^, where six mass spectra were averaged at each wavelength.

### Computational Modeling

Geometries were optimized in the gas phase using density functional theory (DFT) employing the conventional hybrid B3LYP^70,71^ functional with 6-31+G(d,p) basis set. The input geometry was derived from the minimum-energy geometry for the sodium ion complex with 15-crown-5 reported in ref (18), replacing Na^+^ with Co^2+^ and adding the auxiliary H_2_O ligand manually. Higher energy conformations were obtained systematically through relaxed potential energy scans around the OCCO dihedrals of the crown ether and reoptimization of the new geometries. The Gaussian16 program package^72^ was utilized for all computations. The minimum-energy isomer for each of the spin states (doublet, quartet) as well as higher-energy conformers for the quartet states is further optimized at the B3LYP/def2TZVP level. We note that computed geometries and vibrational frequencies at these levels are not significantly different, so that we shall focus our discussion around the B3LYP/6-31+G(d,p) results and relay results for additional levels of theories in the Supporting Information (see Figure S1).

Harmonic vibrational frequencies were calculated for the optimized geometries. No imaginary frequencies were encountered, confirming that the stationary points were true minima on the molecular potential energy hypersurface. Co(II) has a d^7^ electronic configuration, and both doublet (low-spin) and quartet (high-spin) configurations are considered within an approximately octahedral ligand coordination environment (see Scheme 1). Computed frequencies were convoluted with a 15 cm^–1^ (fwhm) Gaussian line shape function to approximately match the observed bandwidth in the IRMPD spectra. Harmonic IR frequencies were scaled^73,74^ by a factor of 0.985 to compensate for anharmonicity and basis set incompleteness.

## Results and Discussion

### Computed Geometries of the Isomers

**Figure 1:**
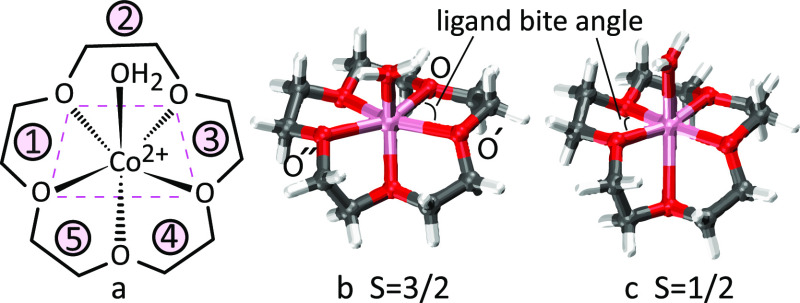
B3LYP/6–31+G(d,p)-optimized geometries of the hydrated Co(II) complex [Co(15-crown-5)(H_2_O)]^2+^ (a) in its quartet (b) and doublet (c) spin multiplicities*.* Chelate ring conformations^79^ are δλδδδ in the order of chelate rings 1–5 (a). Co-O bond length and ligand bite angle (OCoO) listed in Table 1 are indicated.

Figure 1 shows the optimized minimum-energy geometries for the quartet and doublet spin states, and Table 1 summarizes the key structural parameters for both spin states. The complexes show pseudo-octahedral coordination of the six oxygen atoms – five from the crown ether and one from the water ligand – to the central cobalt ion; these structures can also be considered as distorted-square-bipyramid. The key geometrical difference between the two spin states involves the bond lengths of the axial Co–O bonds. For the doublet spin state, these bonds are elongated relative to the equatorial Co–O bonds, whereas in the quartet spin state, the axial Co–O bonds are compressed. The axial elongation^75,76^ of the pseudo-octahedral geometry in the doublet spin state is perhaps related to Jahn–Teller distortion of this spin state in the gas phase.

**Table 1:** Optimized Average Structural Parameters of the Gaseous Complex [Co(15-crown-5)(H_2_O)]^2+,^^b^

	B3LYP/6-31+G(d,p)		B3LYP/Def2TZVP		PW6B95/Def2TZVP	
	*S* = 3/2	*S* = 1/2	*S* = 3/2	S = 1/2	*S* = 3/2	*S* = 1/2
bond length (Å)						
Co–O (axial)^a^	2.092	2.389	2.097	2.393	2.085	2.302
Co–O (equatorial)	2.150	1.996	2.177	2.003	2.144	1.987
						
bond angle (°)						
O–Co–O′ (bite angle)	75.8	78.3	75.7	78.0	76.1	79.1
O–Co–O″	142.3	155.8	142.0	155.5	143.6	157.5

a Average value of crown and water axial Co-O bond lengths.

b Atom labels are shown in Figure 1b.

The axial elongation and equatorial compression upon switching from high-to-low spin state is accompanied by an increase in the bond equatorial O–Co–O bond angles. These observations are consistent with similar six-coordinate complexes^40,77,78^ confirmed by previous magnetic susceptibility measurements including EPR spectroscopy.^50^ High-to-low spin conversion involves transfer of an electron from the e_g_ to the t_2g_ orbital, which often strengthens the metal–ligand bonds also via π back-donation of the metal ion to a vacant π*-orbital of the ligand. Computed bond distances are comparable with relevant X-ray crystallographic data.^50,51,58−60^

Although we realize that all presented calculations are single-reference in nature, we do report the computed energy gap between the two spin states as approximately 120 ± 10 kJ mol^–1^, with the quartet state being preferred. Besides the minimum energy conformer, additional higher energy conformers are computationally characterized (Table 2) for the quartet spin state to verify their existence (*vide infra*). The Co–O bond length of the bound water remains roughly equal (2.07–2.10 Å) for all six conformers as in the solid.^59^ Similarly, the other axial Co–O bond varies between 2.06 and 2.12 Å. These results are consistent with X-ray absorption spectroscopic data (Co–O/N = 1.96 Å for high spin and Co–O/N = 1.88 Å for low spin) determined for a dihydrated Co-complex with phenolic dianion of the Schiff base, *N*,*N*′-ethylenebis(3-carboxysalicylaldimine) ligand,^46^ resembling [Co(15-crown-5)(H_2_O)]^2+^.

**Table 2:** Computed Relative Free Energies (kJ mol^–1^) of the Quartet-State Conformers in the Gas Phase^a^

#	B3LYP/6-31+G(d,p)	B3LYP/Def2TZVP	SP-MP2/6-311+G(2d,2p)
A	0	0	0
B	4.4	3.7	7.0
C	5.0	4.2	7.9
D	7.1	7.4	15.2
E	10.6	10.9	15.4
F	11.7	12.0	18.1

a Single point (SP) energies are calculated using MP2/6-311+G(d,p) on the optimized geometries of the B3LYP/6-31+G(d,p) level

Average ligand bite angles (OCoO) within 15-crown-5 vary between 73.5° and 76.1°. These values are apparently slightly larger than 72 ± 1° determined by the X-ray crystallography for the solid complex, [Co(15-crown-5)(acetonitrile)_2_][CoCl_4_].^60^ However, the equatorial tetra-coordinated plane is evaluated via the dihedral angle of the four O-atoms, indicated by the magenta dashed line in Figure 1a; it varies between 3.4° and 15.9° for the various conformers in the quartet spin state. Interestingly, these four O-atoms form a trapezoid keeping the Co ion nearly in the plane.

### IRMPD Spectra of [Co(15-crown-5)(H_2_O)]^2+^

**Figure 2:**
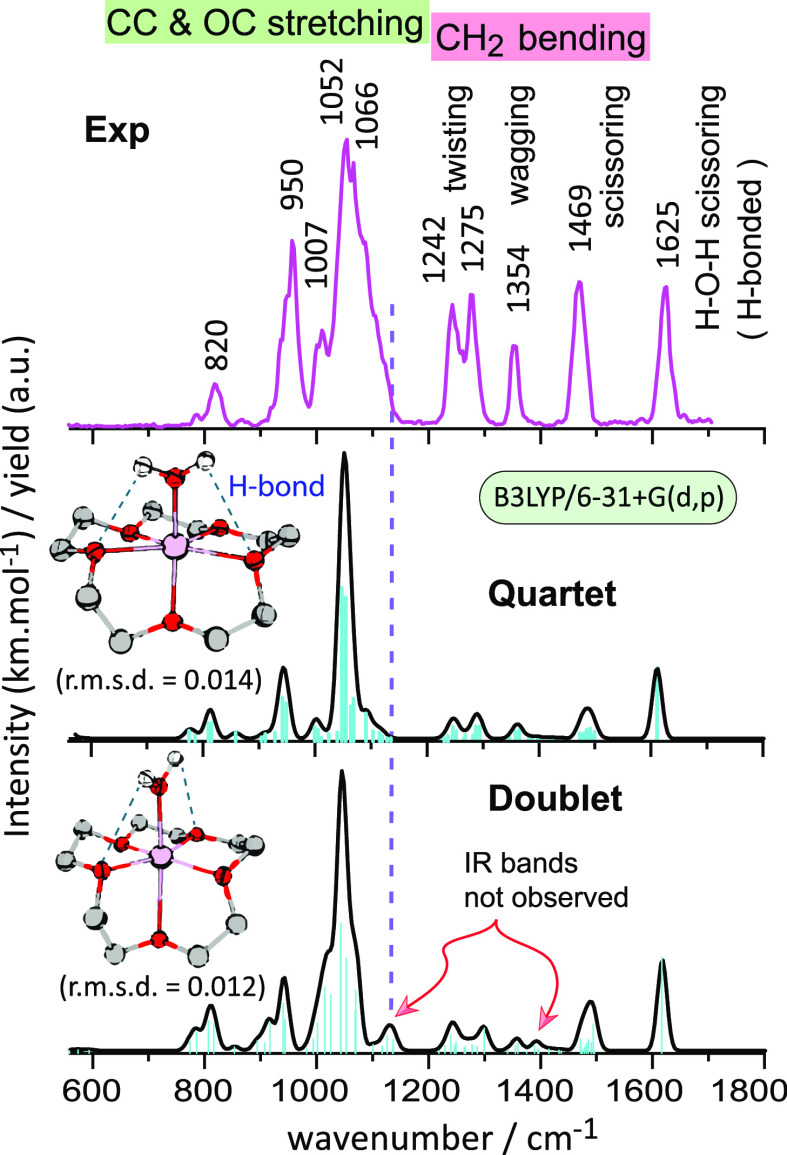
IRMPD spectrum of [Co(15-crown-5)(H_2_O)]^2+^ compared with the calculated IR spectra (black) of the minimum-energy conformers of the complex in its quartet and doublet spin state (B3LYP/6-31+G(d,p) level). The IR spectrum computed for the quartet spin state provides a better match with the experiment than that for the doublet spin states. Computed geometries of this level are merged with that of B3LYP/Def2TZVP level (IR spectra in supplementary information Figure S2) and deviations in atom positions are expressed as rmsd (only CoO_6_ coordination is considered). Approximate vibrational mode characters are indicated in the experimental spectrum.

Figure 2 shows the experimental IRMPD spectrum of [Co(15-crown-5)(H_2_O)]^2+^. Several discrete and well-resolved vibrational bands are observed. At every absorption band, neutral loss of H_2_O is observed as the only fragmentation channel. Figure 2 compares the experimental IRMPD spectrum with calculated linear IR spectra of the complex in doublet and quartet spin states. Despite the similarities of the computed spectra, subtle differences are apparent in terms of the IR band positions and relative intensities. These small IR spectral differences can essentially be attributed to the geometrical differences imposed by the different spin multiplicities.

The highest-frequency IR band observed is at 1625 cm^–1^, which is predicted with a slight red-shift at 1612 cm^–1^. This band is due to the bound H_2_O ligand and corresponds to its localized H-O-H scissoring mode. Both hydrogen atoms form H-bonds with O-atoms of the crown macrocycle, explaining the slight stiffening of this mode compared to gaseous H_2_O, having its H–O–H bending mode at ∼1590 cm^–1^; in the condensed phase, this mode is further to the blue near 1650 cm^–1^, due to the formation of hydrogen bonds.^80^

Band assignments in the 600–1200 cm^–1^ range involve mainly CC and CO stretching vibrations. Most of these vibrations are characteristic for the crown ligand and compare closely to analogous bands reported for Zn and Cd crown complexes, without the auxiliary H_2_O ligand.^35^ The dominant IR band in this range appears broadened (fwhm ≈ 60 cm^–1^) with two apparent maxima at 1052 and 1066 cm^–1^. One of these bands is predicted well at 1052 cm^–1^ for the minimum-energy conformer of the quartet spin state complex. Moreover, the other observed band is convincingly reproduced by multiple higher-energy conformers, rationalizing the broadening of this band (*vide infra*).

Computed normal modes in the range 1200–1500 cm^–1^ are mainly due to CH_2_-bending vibrations. Although differences in the computed spectra for the two spin states are subtle, the predicted IR bands for the quartet spin state complex match closely with the observed spectrum, whereas slight deviations are observed with those computed for the doublet spin state complex in Figure 2 (see arrows). For instance, the calculated band at 1390 cm^–1^ (11 km mol^–1^) is absent while the band near 1360 cm^–1^ with similar intensity (14 km mol^–1^) is present in the experiment. Similarly, the band predicted at 1131 cm^–1^ (28 km mol^–1^) for the doublet spin state is not clearly reproduced experimentally; the shoulder observed on the main band in the experiment resembles more the calculation for the quartet state. This band is due to a mode with axial O–Co–O stretch character and therefore sensitive to the axial elongation of the crown Co–O bond relative to the water Co–O bond, where the two spin states show prominent geometrical differences. We therefore conclude that the experimentally observed complex is in its quartet spin state.

### Presence of Higher Energy Conformers?

**Figure 3:**
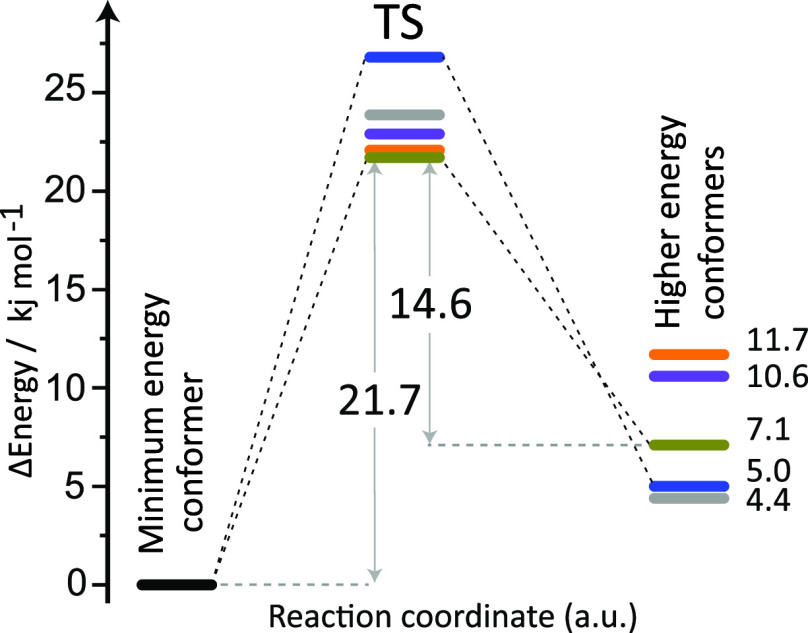
Computed transition state barriers from the lowest-energy conformer to five higher energy conformers for the quartet spin state. The lowest TS barrier and the corresponding thermalization energies in kJ mol^–1^ are indicated (B3LYP/6-31+G(d,p) values).

The 15-crown-5 macrocycle contains five ethylene (−CH_2_–CH_2_−) units that are linked by five oxygen donor atoms. Intrinsically, these constitute rotatable single bonds that facilitate the formation of various conformers of the metal–ligand complex, where relative chelate ring conformations are swapped between λ and δ.^79^ In addition to the lowest-energy geometry, five higher-energy conformers can be generated since there are five ethylene units. Relative energies of these conformers are up to ∼12 kJ mol^–1^ higher than the global-minimum conformer (Figure 3). Calculations at the B3LYP/Def2TZVP and SP-MP2/6-311+G(2d,2p) level confirm the trend (Table 2). To visualize the geometrical differences, the minimum-energy conformer is merged with the higher-energy ones in Figure S3 in the Supplementary Information.

Transition-states (TSs) connecting the various conformers were calculated, revealing relatively low-energy barriers ranging between 22 and 27 kJ mol^–1^. The lowest TS barrier of ∼22 kJ mol^–1^ leads to an alternative conformer at 7.1 kJ mol^–1^. Two conformers at 4.4 and 5.0 kJ mol^–1^ require higher TSs to be accessed from the minimum-energy conformer. From the TS calculations, we extract thermalization barriers — the energy required for the higher-energy conformers to convert back to the minimum-energy conformer — ranging between 15 and 22 kJ mol^–1^. These values suggest that some higher-energy conformers may remain kinetically trapped,^68,81^ reluctant to thermalize back to the global minimum in the ion trap. TS values found here are similar to those recently reported for the [Ni^2+^-hexa-aza-18-crown-6] complex,^39^ and experimental evidence for the kinetic trapping of higher energy conformers was previously reported for crown ether complexes with alkali metal cations.^33^

**Figure 4:**
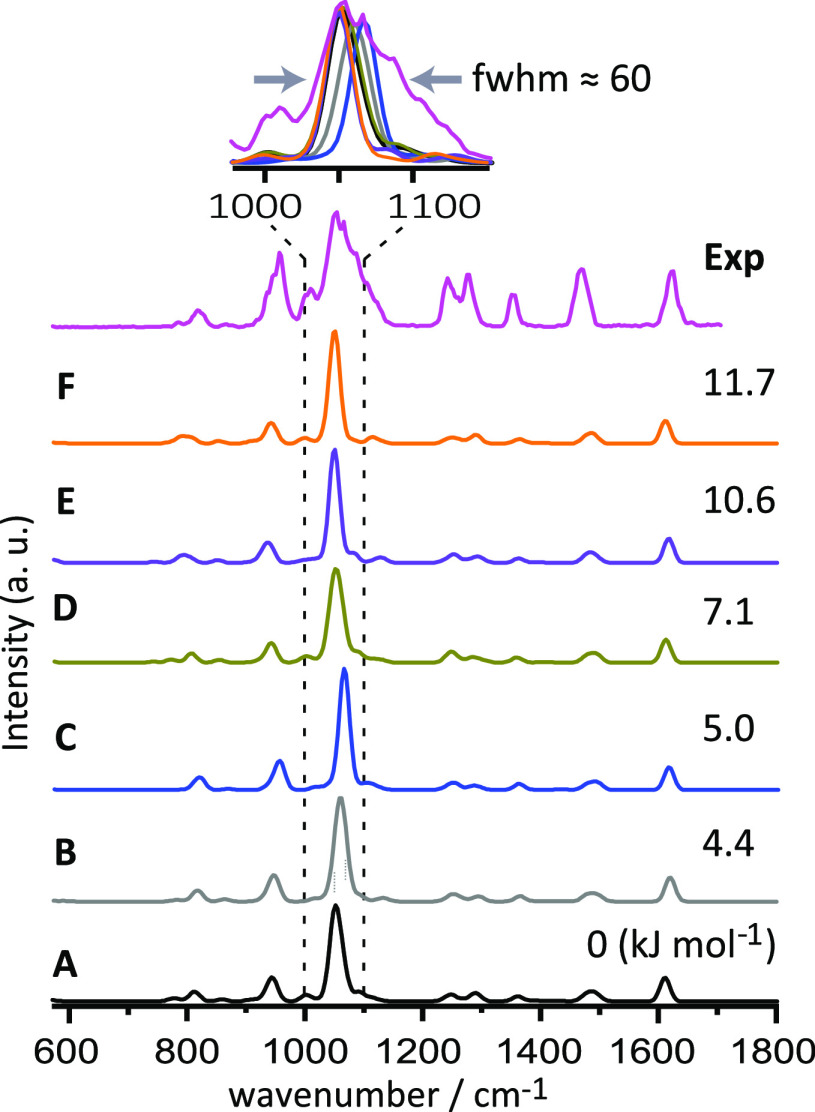
IRMPD spectrum of [Co(15-crown-5)(H_2_O)]^2+^ compared with the calculated IR spectra of its conformers (A–F) in their quartet spin state. All predicted IR spectra and the experimental spectrum are overlaid (top panel) to assess the observed broadening of the main band in the IRMPD spectrum. Gibbs free energies (B3LYP/6-31+G(d,p)) are shown with respect to the minimum energy conformer. Optimized conformers are presented in Figure S3. Results at the B3LYP/def2TZVP level are similar, see Figure S4.

Not only the relatively low energies of the conformers but especially their IR spectral signature suggests the simultaneous presence of multiple conformers. Figure 4 shows predicted IR spectra of the higher-energy conformers along with the measured IRMPD spectrum. As expected, the computed IR spectra of the different conformers are similar, although subtle differences are noted. In particular, the dominant IR absorption near 1050 cm^–1^ due to C–O–C stretching modes shows blue-shifts of up to 14 cm^–1^ relative to this band in the minimum-energy conformer (A). In the second and third conformer (B and C), the blue shift is especially appreciable and the bands in these conformers overlap with the broadened wing of this feature in the experimental IRMPD spectrum, as is more clearly seen in the zoom-in in Figure 4. Overall, the fractional presence of these higher-energy conformers, promoted by their kinetic trapping, may rationalize the observed band profile. Similar arguments may explain the observed broadening of the band near 950 cm^–1^ (see Figure S5). Furthermore, the observed IR band at 820 cm^–1^ is reproduced more closely by conformers A–C than by the other conformers D–F.

If these suggested band assignments are correct, the experimental IR data presented here provide evidence for the presence – and kinetic trapping – of the higher-energy conformers, supporting what is suggested by the conformer relative energies and TS barriers. From our experimental spectrum, it is not possible to derive the relative fractions of higher-energy conformers. A room-temperature Boltzmann distribution based on the DFT energies suggests that about 35% of the ions reside in higher-energy conformers, but this fraction may be higher if kinetic trapping occurs. *Mutatis mutandis,* this appears to be qualitatively in line with the observation of anomalous bond dissociation energies for alkali metal cation crown ether complexes,^17,18,33^ which were attributed to a significant fraction of higher-energy conformers.

## Conclusions

We have measured the IRMPD spectrum of mass-selected [Co(15-crown-5)(H_2_O)]^2+^ in the gas phase to investigate the chelation motif of the complex including the correct electronic structure. According to DFT calculations, Co^2+^ is chelated in a hexa-coordinated fashion by all six O-atoms of the 15-crown-5 and H_2_O ligands. This gives a distorted octahedral configuration with a quartet spin multiplicity. Due to the bound molecular water, the quasi-planar geometry of bare [Co(15-crown-5)]^2+^ (see Figure S1) adopts a distorted geometry. Spectral evidence confirms the presence of strong H-bonds of the protons of the water molecule with crown ether O atoms. Furthermore, the presence of higher-energy conformers is investigated based on their favorable thermodynamics (4–12 kJ mol^–1^), relatively low transition state barriers and, moreover, comparison of the experimental spectrum against computed spectra for all conformers (formed by rotation of −CH_2_–CH_2_– units within the crown ether). Furthermore, the calculated thermalization barriers may favor kinetic trapping of higher-energy conformers. Addition of a single molecular water to the bare [Co(15-crown-5)]^2+^ complex transforms its planar coordination geometry to a square-bipyramidal one in the [Co(15-crown-5)(H_2_O)]^2+^ complex, retaining the high-spin (quartet) multiplicity in the gas phase.

## Supporting Information Available

Computed IR spectra of bare and hydrated [Co(15-crown-5)]^2+^ (Figure S1); computed IR spectra at the B3LYP/def2TZVP level (Figure S2); geometry of six conformers of [Co(15-crown-5)(H_2_O)]^2+^ at the B3LYP/6-31+G(d,p) level (Figure S3); computed IR spectra of conformers at the B3LYP/def2TZVP level (Figure S4); overlaid IR spectra of all conformers (Figure S5); additional data: *xyz* coordinates of optimized geometry of minimum-energy conformer in quartet spin state (PDF)
