# Supplementary material for: Infrared Ion Spectroscopic Characterization of the Gaseous [Co(15-crown-5)(H2O)]2+ Complex
Source: J Phys Chem A. 2023 Aug 18;127(34):7256–63. doi: 10.1021/acs.jpca.3c04241 (PMC10476210; doi:10.1021/acs.jpca.3c04241)
Supplement: Supplementary file 1 — jp3c04241_si_001.pdf [file jp3c04241_si_001.pdf]

Supporting Information

Infrared Ion Spectroscopic Characterization of the Gaseous $[Co(15\text{-crown-}5)(H_2O)]^{2+}$ Complex

Musleh Uddin Munshi[a*], Giel Berden[b], Jos Oomens[b, c*]

[a]Department of Chemistry, Sogang University, Seoul 04107, Republic of Korea, e-mail: musleh@sogang.ac.kr

[b]Radboud University, Institute for Molecules and Materials, FELIX Laboratory, Toernooiveld 7, 6525 ED Nijmegen, The Netherlands, e-mail: j.oomens@science.ru.nl

[c]University of Amsterdam, Science Park 904, 1098XH Amsterdam, The Netherlands

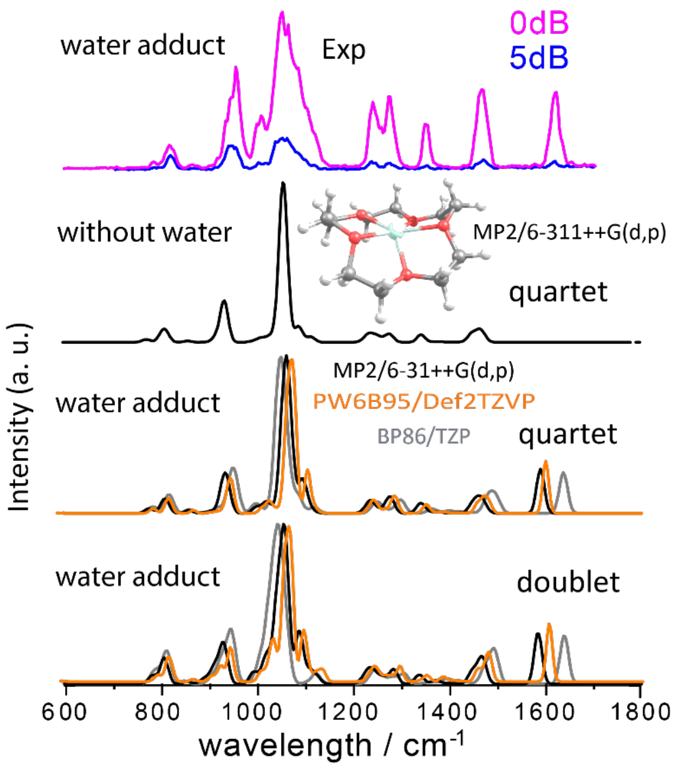

Figure S1 IRMPD spectra recorded for the mass-selected $[Co(15\text{-crown-}5)(H_2O)]^{2+}$ ions are compared with the computed frequencies for bare $[Co(15\text{-crown-}5)]^{2+}$ and (hydrated) $[Co(15\text{-crown-}5)(H_2O)]^{2+}$.

Additional computational modeling

Additional calculations of the complex were performed at different levels of theory. MP2/6-311++G(d,p) was applied for the bare $[Co(15\text{-crown-}5)]^{2+}$ complex in its quartet spin state, and MP2/6-31++G(d,p), BP86/TZP and PW6B95/def2TZVP (suggested by Truhlar et. al.[1]) calculations were performed for $[Co(15\text{-crown-}5)(H_2O)]^{2+}$ in both quartet and doublet spin states. Optimized geometries at the B3LYP/6-31+G(d,p) level were used as the starting geometry for these optimizations. The Gaussian16[2] program package was used for all computations. Harmonic vibrational frequencies were scaled with 0.96 (MP2), 1.03 (BP86) and 0.975 (PW6B95/Def2TZVP), see Fig. S1. Compared with the experimental frequencies, the computed frequencies at these levels are not better than B3LYP.

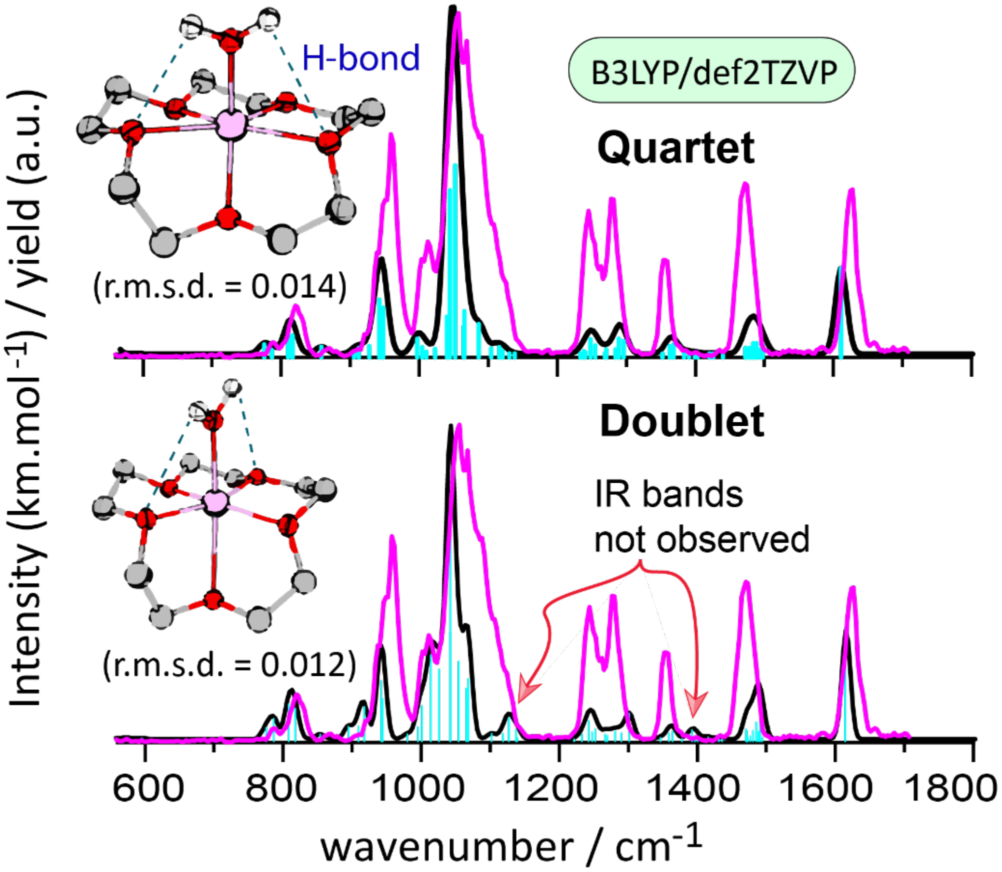

Figure S2 IRMPD spectrum of $[Co(15\text{-crown-}5)(H_2O)]^{2+}$ compared with the calculated IR spectra (black) of the minimum-energy conformers of the complex in its quartet and doublet spin states. Computed geometries of this B3LYP/Def2TZVP level of theory are merged with that of B3LYP/6-31+G(d,p) (Fig. 2 in main text) and deviations in atom positions are expressed as rmsd atom positions in Å (only CoO_6 coordination is considered).

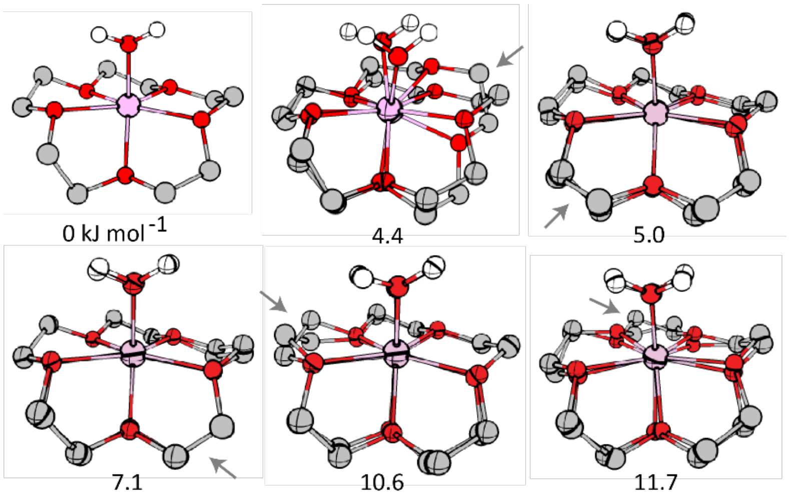

Figure S3 Five higher energy conformers are merged individually with the global minimum-energy isomer. Salient structural differences are indicated with arrows. H atoms are omitted from the 15-crown-5 ether for clarity. Gibbs free energies in kJ mol^{-1} are indicated. Geometries are calculated in their quartet spin state at the B3LYP/6-31+G(d,p) level. Note similar pseudo-octahedral structures were calculated for $[Ni(aza\text{-}18\text{-}crown\text{-}6)]^{2+}$.[3]

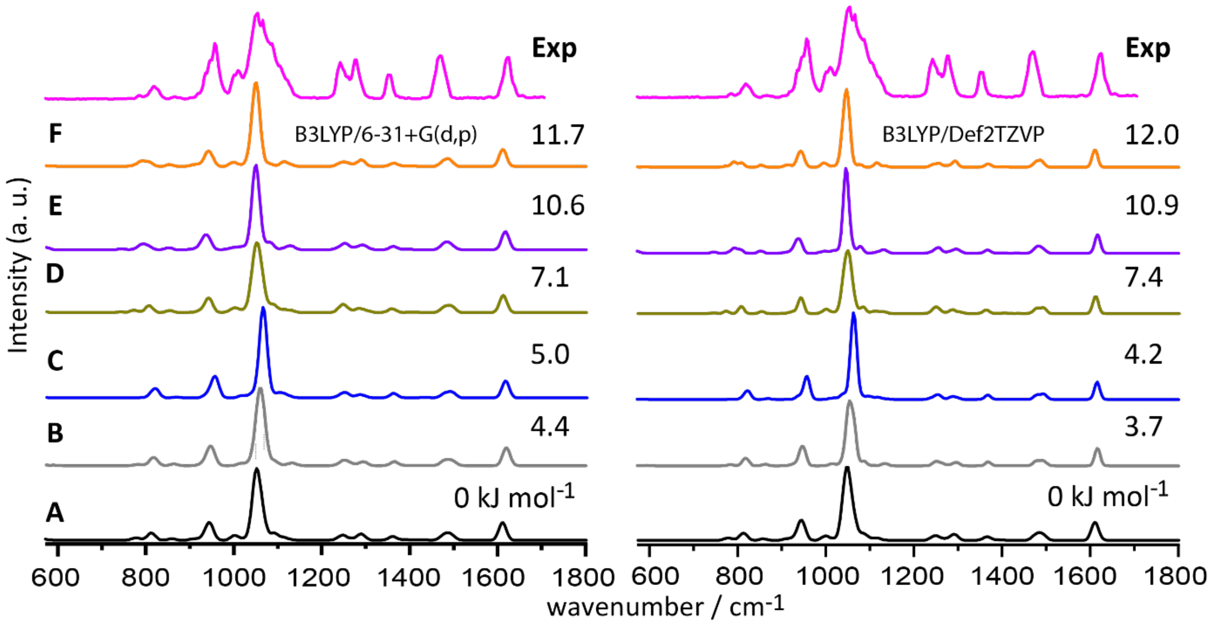

Figure S4 IRMPD spectrum of $[Co(15\text{-}crown\text{-}5)(H_2O)]^{2+}$ compared with the calculated IR spectra of its different conformers (A-F) in their quartet spin state. Relative Gibbs free energies with respect to the minimum energy conformer are shown and give the correspondence to the geometries in Figure S3.

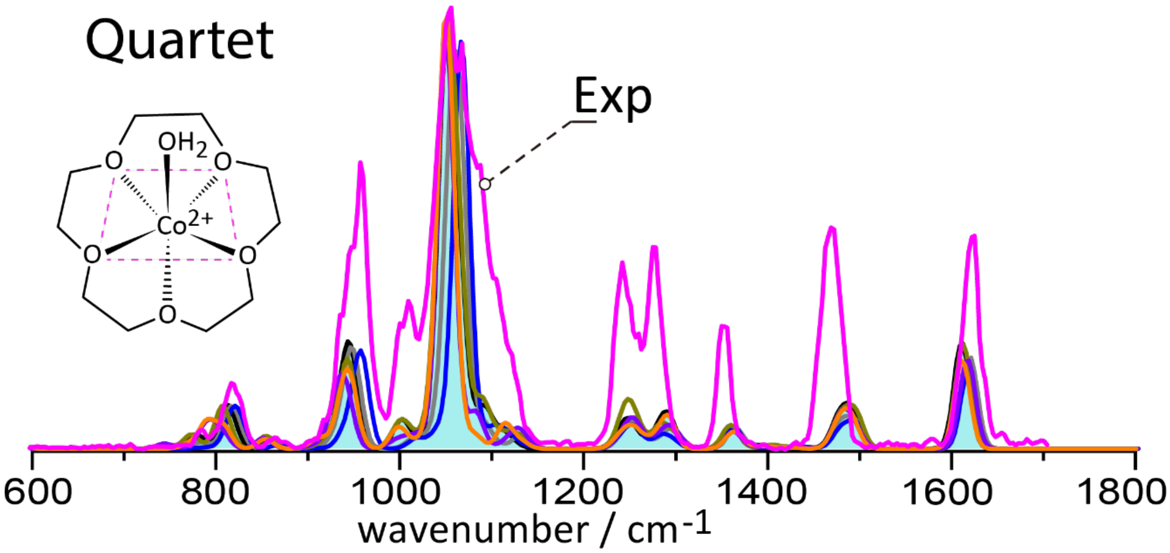

Figure S5 All six predicted IR spectra (colored traces, A-F of Figure 4) using B3LYP/6-31++G(d,p) for the quartet spin state are overlaid with the experimental IRMPD spectrum to assess the broadening of the bands in the IRMPD spectrum. Shaded (cyan) area belongs to the minimum-energy conformer.

References

1) Verma, P.; Varga, Z.; Klein, J.; Cramer, C. J.; Que, L.; Truhlar, D. G. Assessment of Electronic Structure Methods for the Determination of the Ground Spin States of Fe (II), Fe (III) and Fe (IV) Complexes. *Phys. Chem. Chem. Phys.* **2017**, *19* (20), 13049–13069.
2) Frisch, M. J., Trucks, G. W., Schlegel, H. B., Scuseria, G. E.; Robb, M. A.; Cheeseman, J. R.; Scalmani, G.; Barone, V.; Petersson, G. A.; Nakatsuji, H., et al., Gaussian 16, Revision C.01, Gaussian, Inc., Wallingford CT, 2016.
3) Munshi, M. U.; Berden, G.; Oomens, J. Facial vs. Meridional Coordination in Gaseous Ni(II)–Hexacyclen Complexes Revealed with Infrared Ion Spectroscopy. *Phys. Chem. Chem. Phys.* **2022**, *24* (43), 26890–26897.

Optimized coordinates of minimum-energy structure (quartet state)

O	-1.113207000	1.676733000	-0.471412000
C	1.012867000	2.590743000	-1.006156000
H	1.669266000	3.455031000	-0.891107000
H	1.013618000	2.272094000	-2.051732000

C	-0.378389000	2.924344000	-0.522685000
H	-0.357152000	3.367062000	0.476134000
H	-0.870144000	3.609873000	-1.214831000
O	1.479951000	1.504837000	-0.174389000
C	2.843262000	1.075808000	-0.369248000
H	3.526317000	1.901226000	-0.158700000
H	2.976840000	0.761426000	-1.407769000
C	3.057175000	-0.054495000	0.616995000
H	4.007011000	-0.560163000	0.439879000
H	3.054454000	0.323286000	1.638645000
C	2.138476000	-2.052486000	-0.432726000
H	2.449385000	-1.635134000	-1.393648000
H	2.900910000	-2.749962000	-0.080069000
C	0.811423000	-2.749239000	-0.562490000
H	0.842684000	-3.474985000	-1.376178000
H	0.534138000	-3.253116000	0.366429000
O	1.961271000	-0.994835000	0.534638000
C	-1.529452000	-2.210290000	-1.072345000
H	-1.911992000	-1.683269000	-1.945683000
H	-1.514318000	-3.276969000	-1.295796000
C	-2.339000000	-1.918805000	0.174572000
H	-3.398087000	-2.131390000	0.019918000
H	-1.992771000	-2.506834000	1.024305000
O	-0.172767000	-1.727234000	-0.871623000
C	-3.059937000	0.390391000	-0.099259000
H	-4.032945000	0.343323000	0.393135000
H	-3.177130000	0.122591000	-1.151262000
C	-2.466572000	1.770788000	0.025254000
H	-3.034513000	2.481603000	-0.577711000
H	-2.445219000	2.117571000	1.061843000
O	-2.148007000	-0.533753000	0.544222000
Co	0.009807000	0.044986000	0.248777000
O	-0.097227000	0.166306000	2.333405000
H	-0.937769000	0.000283000	2.780850000
H	0.613387000	-0.035167000	2.956161000
